# Emotional intelligence and academic motivation in primary school students

**DOI:** 10.1186/s41155-022-00216-0

**Published:** 2022-05-27

**Authors:** Julia Arias, Jorge G. Soto-Carballo, Margarita R. Pino-Juste

**Affiliations:** GIES-10 research group, Universidade de Vigo, Tui, Pontevedra Spain

**Keywords:** Emotional intelligence, Academic motivation, School task, Primary school

## Abstract

The role of emotions in the educational context is one of the lines of research that has generated most interest in recent years. This study explores the level of emotional intelligence (EI) and motivation towards studying of primary school (PS) students, as well as the relationship between both variables. For this, a quasi-experimental design has been used with an accidental sample of 541 students from public centers in the province of Pontevedra (Spain). The instruments used were a School Motivation Scale and an EI questionnaire for primary school students, based on the five areas of Goleman EI. The results maintain a mid to high level of EI in all of the factors (self-conscience, self-control, emotional use, empathy and social skills) and a good level of academic motivation. Therefore, they show a positive and significant correlation of both variables. Girls have a higher emotional intelligence index and there is no difference in academic motivation in terms of gender. Based on these results, it is suggested to implement programs that consolidate emotional competences given their importance in the psychoevolutionary development of students and their relationship with academic motivation.

## Theoretical framework

Education in Spain, and therefore the primary education (PE) stage, has been exposed to numerous legislative and structural reforms in recent decades. Currently, this stage is focused on the acquisition of competency development, which includes the learning of personal, intellectual, social, and emotional skills. The ultimate goal is that students can learn and develop these skills and abilities that allow them to face their future and adapt to the changing situations of the knowledge society (Codero & Manchón, 2014; Merchán et al., 2014; Asensio et al., 2015). In this sense, more and more voices are emerging that question the exclusivity of rational intelligence as the most influential factor in achieving academic and personal success, understanding that there are other variables that should also be taken into account, such as emotional intelligence (EI) and motivation (Ferrándiz et al., 2012; Filella et al., 2014; Bisquerra et al., 2015; Pulido & Herrera, 2017; Rebollo & de la Peña, 2017; Puertas et al., 2020).

The key lies in the harmony between thoughts and emotions, and education is the main instrument to achieve this harmony, to achieve an integral development of people, not only at a cognitive level, but also in the social and emotional aspect. New times demand a change in academic slogans, bringing emotional education closer to formal education (Aguadez & Pantoja, 2015; Valdés & Gutiérrez, 2018; Solé, 2020). Hence, one of the most preeminent purposes of education should be to train emotionally intelligent students, understanding emotional intelligence as “the ability to access and generate feelings that facilitate thoughts” (Mayer & Salovey, 1997, 10).

Historically, in Western culture, human beings have always been considered rational, leaving the emotional world in the background. But this way of understanding the human being is incomplete, since emotions are present in all the acts and moments of our life. As biologist Maturana (2001) points out, we live in a culture that devalues emotions in favour of reason. By defining the human being as a rational being, we do not realize that there is no human action without an emotion that grounds it as such and makes it possible as an act.

One of the most forceful critics of the traditional model of conceiving intelligence was Gardner (1983). In his work Frames of Mind he questions the famous “IQ”, or “intellectual quotient”, invented by Binet in 1905, since it measures only two of the eight intelligences we possess, linguistic and logical-mathematical intelligence, and reformulates the concept of intelligence through the theory of multiple intelligences, which establishes that we possess different types of intelligence and each one is relatively independent of the others.

For Gardner (1983), intelligence is a genetic potential that has to be developed by education and that manifests itself in eight different fields: linguistic, logical-mathematical, spatial, kinesthetic-bodily, musical, natural-ecological, intrapersonal, interpersonal. Intrapersonal refers to the emotional life of each person, the knowledge of their feelings, the management of their emotions, and the ability to direct their own behavior. The interpersonal is built on the ability to establish good relationships between people, to understand others, to understand their moods, temperaments, intentions, etc.

The first publications that appeared on emotions and the importance of learning to know and regulate them, made many statements about the positive influence of EI in the classroom. The only drawback was that all these assertions were not supported by empirical data showing that EI skills and competencies had real and positive repercussions in the school and personal life of students (Extremera & Fernández-Berrocal, 2004).

Since the 1990s, numerous studies have shown that our feelings and emotions influence our thoughts. Both in the field of psychology (Mayer & Salovey, 1990; Goleman, 1996; Fernández-Berrocal et al., 2004; Bar-On, 2006; Mayer et al., 2008; Filella et al., 2014) as well as important biologically based works (Hamann et al., 2020; Sánchez & Román, 2004), developed with the help of new technologies (functional magnetic resonance imaging or positron emission tomography-PET), made possible great scientific advances in the field of affective neuroscience and more specifically in the field of EI (Trujillo & Rivas, 2005; Punset, 2010). Therefore, the concept of EI is acquiring, over the years, a solid scientific basis. In this sense, a short excerpt from Goleman (1996, 67) states that:

“The existing connections between the amygdala (and the limbic structures related to it) and the neocortex constitute the center of gravity of the existing struggles and cooperation treaties between the heart and the head, between thoughts and feelings. This neural pathway would explain why emotion is so fundamental to effective thinking, both to make intelligent decisions and to enable us to think clearly”.

We have to clarify here the contradictions between the explanations of neuroscientists and educators or social scientists. For the former in general, emotions appear caused by needs of the organism triggered internally or by external events. Therefore, the social and environmental environment is only an external trigger of emotion. To understand it, it is not necessary to analyze the external triggers but rather what happens in the brain (Damasio, 2018).

From the field of social sciences, there are currently two main models of EI, the “skills model” (Mayer & Salovey, 1990, 1997), which bases the EI construct on emotional information processing skills, and the “mixed model” (Goleman, 1996), which combines emotional skills with personality traits such as self-esteem, persistence, optimism, frustration tolerance or self-motivation (García-Fernández & Giménez-Mas, 2010; Fernández-Berrocal et al., 2012). In our country, the latter is the most widespread model due to the publishing success of Goleman’s bestseller (Fernández-Berrocal & Extremera, 2005).

The other variable that we are going to analyze in this study is motivation, we can define it as a multidimensional process (formed by biological, cultural, social, learning and cognitive aspects), which is based on the existence of a motive or reason that drives a subject to initiate, develop, and complete a behavior (Burón, 2006). However, it should be borne in mind that, faced with the same situation, each person does not respond or motivate in the same way, which leads us to deduce that there are different types of motivation. Ryan and Deci’s model (2000) distinguishes between intrinsic motivation, where the subject considers the action interesting and satisfying in itself; extrinsic motivation, external to the individual, where the reason for acting is some consequence (gaining a reward or avoiding punishment); and demotivation or a state of absence of motivation.

Intrinsic motivation constitutes one of the psychoeducational factors most involved in school learning and can be considered as the key piece of deep and lasting learning (Barrientos, 2011; Camacho-Miñano & Del Campo, 2015; Morilla-García & García-Jurado, 2019). If students are motivated, if they are interested in understanding what they are studying and acquiring the knowledge and skills, they concentrate more on what they are doing, persist in the search for solutions to the problems they encounter and devote more time and effort than students who lack adequate motivation.

Currently, motivation for task performance continues to be one of the most researched variables linked to the student body (González et al., 2009; Barca et al., 2011; Barrientos, 2011; García-Señorán et al., 2015; Usán & Salavera, 2018; Barreto-Trujillo & Álvarez-Bermúdez, 2020).

These studies attempt to explore the reasons why primary and secondary school students are involved in academic tasks or, on the contrary, show a lack of interest in them. Most of the studies agree that extrinsic motivations are the most prevalent (a better job in the future, grades, rewards, avoidance of punishment, or encouragement from significant people). While intrinsic motivations (learning new things and enjoying activities) occur to a lesser extent. We must bear in mind that motivation is affected by learning experiences and is not a fixed quality of the individual (Tohidi & Jabbari, 2012). In addition, a strong and positive relationship has been established between emotional intelligence and motivation especially if this extrinsic motivation and amotivation (Sontakke, 2016; Usán & Salavera, 2018); although studies in the context of Primary Education are scarce (Ferrándiz et al., 2012; Clarke et al., 2014). Most of them have been conducted with samples of middle school and university students (Durán et al., 2006; Domínguez-Alonso et al., 2016; Lomelí-Parga et al., 2016; Usán & Salavera, 2018). There are also some related to specific fields such as music, language teaching or physical education (López, 2013; Conde & Almagro, 2013; Cera et al., 2015; González-Peiteado et al., 2016; Fierro Suero et al., 2019).

Regarding the differences in emotional intelligence by gender and age, there is still an important controversy since in some studies women manage emotions better (Extremera et al., 2006; Billings et al., 2014; Rebollo & de la Peña, 2017), while other studies have shown that children seem to have a higher EI in some constructs (Filella et al., 2014; Petrides, 2016). Regarding age, it seems that as the individual gets older, their emotional control improves (Karma & Maliha, 2005; Pulido & Herrera, 2017; Rebollo & de la Peña, 2017).

There is also controversy regarding the differences in academic motivation by gender and age. Girls tend to have higher academic motivation than boys and there are no differences with respect to age (Marumo et al., 2019). But there are multiple differences according to the motivational factors (intrinsic or extrinsic) that are evaluated (Cerezo & Casanova, 2004; Torrano & Soria, 2017; Pino-Juste et al., 2021). Women obtain higher scores in educational aspirations, expectations and goals (Delgado et al., 2010; Ramudo et al., 2017). However, boys often attribute successes to internal causes while failure is attributed to external causes (Smith et al., 2002). In addition, the age groups where the studies are carried out tend to have a small range and are concentrated in a specific population (childhood, adolescence, university student).

## Method

This research used a quasi-experimental design from an interpretive approach (Hernández et al., 2006). It is not, therefore, a random selection of participants, but rather we work with groups already accidentally constituted. Moreover, there is no manipulation of variables and there are no control groups. This approach uses data analysis to answer research questions and test previously established hypotheses, and relies on numerical measurement and statistics to establish patterns of behavior in a population.

The objectives of this research focus on (a) identifying the level of school motivation and EI of PE students; (b) analyzing the correlation between the constructs EI and motivation to study and task performance of PE students; and (c) establishing the association between these constructs and the independent variables (gender, grade, and age).

Based on these objectives, the following hypotheses were proposed: (a) PE students have a good level of EI and a high degree of school motivation; (b) there is a high relationship between EI and school motivation; (c) the level of EI is higher in girls; (d) girls have greater school motivation; (e) 6th grade students have a higher level of motivation than 5th grade students; and (f) EI and school motivation levels increase as age increases.

### Participants

The sample is made up of 541 students in 5th and 6th grades of Primary Education, belonging to several public schools in the province of Pontevedra (Spain), aged between 10 and 12 years, of whom 270 are boys and 271 girls, 51.2% in 5th grade and 48.8% in 6th grade; therefore, it is a very homogeneous sample in terms of gender and grade.

### Instruments

Taking into account that this is a study with a quantitative methodology, we used standardized questionnaires for data collection. A scale to measure the level of EI by Chiriboga and Franco (2001), based on Goleman’s (1996) five areas of EI: self-awareness, self-control, self-motivation or emotional achievement, empathy, and social skill and the school motivation scale by Barrientos (2011) to quantify motivation towards study and the execution of school tasks.

To test the reliability of the questionnaires used in this study, the internal consistency of each of them was calculated by means of Cronbach’s Alpha. The coefficient obtained in the Chiriboga and Franco (2001) test, which consists of 60 items, is .871 and in the School Motivation Scale of Barrientos (2011) (SMS), which consists of 18 items, is .806. Thus, according to the recommendations of George and Mallery (2003), the reliability of these scales is very high (> .8).

### Procedure

To initiate the data collection procedure, the management team and the guidance department were contacted in order to explain the purpose and scope of this research and to request their collaboration and participation. After their consent, the families were informed of the research study by means of a circular letter from the center, in which they were asked for permission to apply the questionnaires. All the questionnaires were completed by the same researcher in the presence of the teacher of each classroom. The explanatory instructions for completing the questionnaires were the same in all classrooms. We also provided information about our research work and clarified doubts so that they could complete the instrument without difficulty. We insisted on the anonymity of the answers and that they should be answered as rigorously and seriously as possible, reading all the items carefully.

It is worth mentioning the receptiveness of all students, teachers, and families who were asked to collaborate. Confidentiality was scrupulously respected. The questionnaires were anonymous, so the identity of the students was always protected. The only personal data requested were age, grade and sex. Regarding the ethical aspects of the research, it was carried out following the deontological standards recognized by the Declaration of Helsinki (review Fortaleza-Brazil, October 2013) and in accordance with the EEC Good Clinical Practice Recommendations (document 111/3976/88 of July 1990) and the current legal regulations governing research.

### Data analysis

In order to decide on the statistics to be used in the data analysis, normality tests were calculated for the SMS and the EI test. The Kolmogorov-Smirnov contrast statistic for the School Motivation variable takes the value of .054, so it does not allow us to reject the null hypothesis of normality for significance levels below .195. The same occurs with the variable EI; in this case, the value of the contrast statistic is .035, for significance levels below .162, which also represents an indication of normality of the population (Table 1).

**Table 1:** Normality of the motivation scale and EI test

	Kolmogorov-Smirnov		
	Statistics	*N*	Sig.
SMS	.054	541	.195
EI	.035	541	.162

*SMS* School motivation scale, *IE* Emotional intelligence

Therefore, the descriptive statistics of central tendency (mean) and dispersion (standard deviation) of each of the study variables are used for data analysis. Bivariate correlations are also analyzed using Pearson’s correlation coefficient between the study variables (EI and school motivation). In the case of EI, we take into account the different components or factors that make it up, to see which have a higher correlation with each other, how each factor correlates with total EI and which has a higher correlation with school motivation. In addition, Student’s *t*-means difference and ANOVA statistics were used to establish the association between the independent and dependent variables.

The SPSS statistical analysis software, version 26.0, was used for data analysis. We used a significance level of .05.

## Results

The descriptive statistics that we obtained for each of the variables in the sample are shown in Table 2. According to these data we can say that the mean of school motivation is 1.5, with a minimum value of 0.39 and a maximum value of 2.00. The mean of total EI is 132.22, the minimum value of this variable is 51.00 and the maximum value is 178.00. We can also extract that the EI factor with the highest mean value is social ability ($\overline{\mathrm{x}}$ = 28.02), followed by emotional achievement ($\overline{\mathrm{x}}$ = 27.54), empathy ($\overline{\mathrm{x}}$ = 26.56), and self-awareness ($\overline{\mathrm{x}}$ = 26.40); the EI factor with the lowest mean value is self-control ($\overline{\mathrm{x}}$ = 23.73). The minimum value of each of the EI components or factors is 6 and the maximum is 36.

**Table 2:** Descriptive statistics

	*N*	Mínimum	Máximum	Mean	SD
Motivation	541	.39	2.00	1.50	.306
EI-self-awareness	541	12.00	36.00	26.40	4.681
EI-self-control	541	6.00	36.00	23.72	4.654
EI-emotional	541	7.00	36.00	27.53	4.880
EI-empathy	541	7.00	36.00	26.56	4.704
EI-social skill	541	9.00	36.00	28.01	4.846
EI total	541	51.00	178.00	132.22	19.047

According to the correlation table (Table 3), we can see that all are significant (less than .05) and positive, i.e., direct (as one increases, so does the other). Moreover, in general, we can affirm that there are medium–high correlations between the variables studied. Among the EI components, the highest correlation is between “empathy” and “social skills” (*r* = .835). In contrast, the lowest correlations are between “self-control” and “empathy” and between “self-control” and “social skills”; (*r* = .492) and (*r* = .564), respectively. If we look at the correlations between the different components of EI with “total EI”, we observe that they present a really high magnitude. The highest of these is the one found between “total EI” and “emotional achievement” or “self-motivation” (*r* = .854), if we are talking about intrapersonal EI; and the correlation between “total EI” and “social ability” (*r* = .818), if we are referring to interpersonal EI. But closely followed by the other factors that make up EI, “self-awareness” (*r* = .823); “self-control” (*r* = .783), and “empathy” (*r* = .806). Finally, we observe that the correlation between “school motivation” and “total EI” is (*r* = .857), which is a high correlation between the two main variables of the research.

**Table 3:** Results of Pearson’s correlations between the different factors (*N* = 541)

	MOT.	AWAR.	CONT.	EMOT.	EMP.	S-SKILL
AWAR.						
*r*	.786 (^a^)					
Sig.	.000					
CONT.						
*r*	.612 (^a^)	.644 (^a^)				
Sig.	.000	.000				
EMOT.						
*r*	.848 (^a^)	.780 (^a^)	.762 (^a^)			
Sig.	.000	.000	.000			
EMP.						
*r*	.567 (^a^)	.698 (^a^)	.492 (^a^)	.627 (^a^)		
Sig.	.000	.000	.000	.000		
S-SKILL						
*r*	.650 (^a^)	.582 (^a^)	.564 (^a^)	.652 (^a^)	.835 (^a^)	
Sig.	.000	.000	.000	.000	.000	
EI-TOT						
*r*	.857 (^a^)	.823 (^a^)	.783 (^a^)	.854 (^a^)	.806 (^a^)	.818 (^a^)
Sig.	.000	.000	.000	.000	.000	.000

*MOT* School motivation, *AWAR* Self-awareness, *CONT* Self-control, *EMOT* Emotional exploitation, *EMP* Empathy, *H-SOC* Social skills, *IE-TOT* Total emotional intelligence

^a^The correlation is significant

With regard to the association between the gender variable and school motivation, there are no significant differences between boys and girls (*t* = − 1.029, *p* = .304 > .05).

However, we can observe differences with respect to the EI variable (Table 4). If we look at “total EI”, we see that there are significant differences between the means, being higher for girls (*t* = − 2.980, *p* = .003 < .05). If we analyze the results by EI components, we can confirm that in “self-control” there are no significant differences in terms of gender (*t* = − 1.752; *p* = .08 > .05). The other EI components “self-awareness”, “self-motivation”, “empathy”, and “social skills” show significant differences in favor of girls. In this table we can see that the greatest differences occur in “empathy” and in “emotional achievement or self-motivation”.

**Table 4:** Student’s *t* results for the association between gender and the different EI factors

Dependent variables	Gender	*N*	Mean	*t*	Sig. (bilateral)
EI self-awareness	Child	270	25.92	− 2.385	.017
	Girl	271	26.88		
EI self-control	Child	270	23.388	− 1.752	.080
	Girl	271	24.08		
EI emotional	Child	270	26.98	− 2.662	.008
	Girl	271	28.09		
EI empathy	Child	270	26.01	− 2.762	.006
	Girl	271	27.121		
EI social skill	Child	270	27.52	− 2.406	.016
	Girl	271	28.52		
EI total	Child	270	129.79	− 2.980	.003
	Girl	271	134.64		

As for the relationship between the EI variable and the course variable, there are no significant differences between the 5th and 6th grades (*t* = .102, *p* = .919 > .05). The same occurs with respect to the association between the course variable and school motivation (*t* = .326, *p* = .745 > .05).

To complete the previous results, we analyzed the interaction of the variables EI and school motivation with age as a nominal variable. In Table 5, we observe that there are significant inter-group differences between students in group 1 (10 years) and group 3 (12 years), (*p* = .012 < .05) and between students in group 2 (11 years) and group 3 (12 years), (*p* = .035 < .05), with the level of motivation being higher in younger children.

**Table 5:** Results of ANOVA between the different age groups and the Bonferroni post hoc test for school motivation and EI

V. I	Factor	*N*	Mean	*F*	Sig.	Bonferroni
Motivation	1 (10 years)	246	1.5198	4.306	.014	10 years–12 years = .012 11 years–12 years = .035
	2 (11 years)	275	1.5028			
	3 (12 years)	20	1.3196			
	Total	541	1.5043			
Emotional intelligence	1 (10 years)	246	132.562	3.905	.025	10 years–12 years = .017 11 years–12 years = .024
	2 (11 years)	275	132.557			
	3 (12 years)	20	123.950			
	Total	541	132.227			

In the same way, we analyzed the variable EI as a function of age. We observed that there are indeed significant differences between the group of children in group 1 (10 years) and group 3 (12 years), since *p* = .017 < .05; and between the students in group 2 (11 years) and group 3 (12 years), whose bilateral significance is *p* = .024 < .05.

## Discussion and conclusions

According to the results obtained, we can consider that PE students have a good motivational level and a medium-high level of EI.

As for the EI components, they present higher mean in those referring to social skills, emotional achievement, empathy and self-awareness, and slightly lower in the self-control factor.

In view of the results obtained, we can also affirm that there is a high degree of correlation between the main variables of the EI study and school motivation. Therefore, these results support our main working hypothesis and we can confirm that students with high levels of EI are more motivated to study and perform school tasks. There is not abundant scientific evidence on this relationship in primary school children. However, in some works (Ferrándiz et al., 2012; Conde & Almagro, 2013; López, 2013; Cera et al., 2015; Domínguez-Alonso et al., 2016; Usán & Salavera, 2018), carried out with samples of Secondary School or Conservatory of Music students, it can be seen that the correlation between EI and school motivation is significant, although in a more modest way than in our study.

We can also say that the results obtained verify positive and high correlations between the factors that make up EI, as well as between each of them and total EI. So they coincide with the studies of other authors (Ferrándiz et al., 2012; López, 2013; Lomelí-Parga et al., 2016; Domínguez-Alonso et al., 2016; Pulido & Herrera, 2017; Usán & Salavera, 2018; Broc, 2019), who interpret EI as a management process of multiple factors that act together complementing and converging in the same direction. That is, students with greater EI capacity are more skilled in emotional perception, understanding and regulation, which allows them to put into practice emotional harnessing, empathy, and social skills.

Regarding the interaction of the gender variable with school motivation, we have to say that there are no significant differences between the means of boys and girls, so our research hypothesis, which considered girls to have a higher level of academic motivation than boys, is not fulfilled. However, in the case of EI, we can observe statistically significant differences between the male and female genders in favor of the latter. Therefore, we can conclude that, according to this study, PD girls show a better management of emotional competencies than boys, thus fulfilling our hypothesis.

The gender differences evidenced in this work are also observed in other research conducted in child, pre-adolescent or adolescent populations (Ogundokun & Adeyemo, 2010; DiPrete & Jennings, 2012; Billings et al., 2014; Pulido & Herrera, 2017; Rebollo & de la Peña, 2017). However, in other studies consulted, differences are found in favor of women, in the so-called social competencies or interpersonal intelligence, while men present better results in intrapersonal intelligence (Soriano & Osorio, 2008; Ferrándiz et al., 2012; Filella et al., 2014). In this sense, most authors agree that other variables such as age and personal circumstances should be examined before concluding that gender is a determinant in people’s EI.

With respect to grade level, the results show that there are no significant differences in the levels of motivation and EI between students in the 5th and 6th grades. Therefore, our research hypothesis, which predicted better results in the last year of PD, is not fulfilled.

However, if we take into account the interaction of age on the EI and school motivation variables, we observe a lower mean of school motivation and EI in 12-year-old students compared to 10- and 11-year-old students (who have very similar means). In our case, it is necessary to take into account that these differences may be due to the fact that these are repeating students who have lower mean levels of motivation and EI. In order to obtain conclusive information on the influence of age, we should have a sufficient age range to allow us to make comparisons. Therefore, it would be interesting to extend the age range of the participants in future research, such as the first years of secondary school, which would allow us to consolidate these results.

Although it is true that in some studies it is observed that as the age of the participant’s increases, the results show a significant decrease in the scores on the EI scale (Karma & Maliha, 2005; Ferrando, 2006). According to Ferrándiz et al. (2012), it can be interpreted as meaning that the younger ones do not have as much need to apply their abilities or skills in the emotional and social area, since they are linked to the family context, which normally protects and regulates them. It is as they grow up and discover new contexts that they go through a period of tension that produces feelings of confusion and low levels of self-confidence that can lead to low levels of EI. Other studies, however, coincide in stating that a progressive increase is observed in the EI levels as age and cognitive development advance (Aguadez & Pantoja, 2015; Pulido & Herrera, 2017; Rebollo & de la Peña, 2017).

Therefore, we can conclude that emotional intelligence influences motivation towards school tasks and both in the academic performance of the student body (Ogundokun & Adeyemo, 2010; Mavroveli & Sánchez-Ruiz, 2011; Qualter et al., 2012; Fernandez et al., 2012; Kumar et al., 2013; Essays, 2013; Lomelí-Parga et al., 2016; Sontakke, 2016; Usán & Salavera, 2018; Broc, 2019). Spain is one of the EU countries with the highest number of early school leavers and our school performance rates in international tests are not too satisfactory in terms of the resources allocated to education (OECD, 2019). We consider, based on the results of this research, that establishing EI programs at early ages in schools can contribute to the improvement of social and personal skills that allow students to know themselves better, regulate their emotions and abilities, as well as maintain motivation towards school work. We should, therefore, be committed to the curricular integration of EI in the PE stage. The treatment of EI from the Tutorial Action Plans or from the different areas of knowledge should serve us as a starting point for this integration (Rebollo & de la Peña, 2017; Usán & Salavera, 2018; Broc, 2019).

We have to take into account that cognition and emotion act in the same area of the brain, therefore, teaching how to manage emotions means that girls and boys will be more motivated to learn. The objective is to favor the development of emotional competencies, through their explicit integration into the didactic program. In other words, educating for life, bringing the school closer to daily life. Emotional competences are not acquired if they are not worked directly in the classroom through the key competences and the different tasks and activities. If we propose tasks that awaken the interest and curiosity of students, that allow them to develop creativity and critical and reflective thinking, that challenge them, that are carried out in a cooperative way and with the support of information and communication technologies, we are getting students emotionally involved in learning. This model of education contributes to the integral formation of students, allowing them to acquire the necessary skills and tools to face the challenges of the twenty-first century.

## Limitations of the study

There are reasons to be cautious about generalizing the results obtained in this study. Although the sample is sufficiently large, the use of a cross-sectional and quantitative design may bias the results obtained. It is necessary to continue conducting future research that will allow us to endorse these results in different cultural contexts and, as we have already suggested, broadening the age range. In addition, it would be important to design longitudinal studies to determine the evolution of EI and school motivation, as well as the possible interaction of the variables studied with repercussions on the school and personal life of students, and qualitative studies to establish consolidated scientific evidence on this topic. Nevertheless, we consider that the results of this research work may be indicative of the tendency of PE students’ thinking and perception of EI and motivation to study and perform school tasks.

## Data Availability

Data sharing is not applicable to this article.
